# Diabetes associated with HNF1B: beyond Occam’s razor—A case report

**DOI:** 10.1007/s00592-025-02472-9

**Published:** 2025-02-20

**Authors:** Carolina Sager-La Ganga, Clara Solà, Karen Castillo, Carme Figueredo, Ignacio Conget

**Affiliations:** 1 Department of Endocrinology and Nutrition, Hospital Universitario de La Princesa. Instituto de Investigación Sanitaria de La Princesa, Universidad Autónoma de Madrid, 28006 Madrid, Spain; 2 Department of Endocrinology and Nutrition, Hospital Clinico de Barcelona, 08036 Barcelona, Spain

## Introduction

Monogenic diabetes is a rare cause of diabetes in the population, accounting for an estimated 1–5% of diabetes cases. Variants in the hepatocyte nuclear factor 1 beta (*HNF1B*) gene represent approximately 1–6% of monogenic diabetes causes [1]. The *HNF1B* gene, located on chromosome 17q12, plays a fundamental role in organogenesis, primarily of the kidney, pancreas, liver, and genitourinary system. Pathogenic variants of *HNF1B* are diverse and include intragenic alterations, insertions, and deletions affecting only parts of the gene, among others. However, the most common occurrence, present in about half of the patients, is microdeletions on chromosome 17q12 that affect the entire gene and include neighboring genes (e.g., *ACACA* and *LHX1*) [2]. *HNF1B* haploinsufficiency leads to a diverse and highly variable clinical phenotype spectrum, potentially leading to renal cysts and diabetes syndrome (RCAD) and, when associated with neuropsychiatric abnormalities, 17q12 deletion syndrome. The most common clinical manifestation involves structural renal anomalies, observed in 90% of cases (most frequently renal cysts), with chronic kidney disease developing in approximately 50% of patients. Among extrarenal manifestations, diabetes is the most frequent (50–80%), with an average diagnosis age of 24 years, followed by genitourinary tract malformations, hypomagnesemia, hyperuricemia, and altered liver profiles, among others [3, 4].

This report presents a clinical case of diabetes associated with *HNF1B* in a patient who was initially enrolled in a clinical trial for type 1 diabetes (T1D) and received immunosuppressive treatment for four years. At the time, knowledge of monogenic diabetes was limited, leading to diagnoses that did not account for these genetic forms. This case also highlights the broad phenotypic spectrum of these genetic alterations and emphasizes the importance of considering this diagnosis even in the absence of a clear family history.

## Case Report

A 49-year-old female has been under outpatient follow-up at our center’s Endocrinology clinic since 1994 (age 19) due to diabetes mellitus initially classified as type 1, managed with insulin therapy. Her family history is negative for monogenic diabetes; her mother and paternal grandfather were diagnosed with type 2 diabetes mellitus at an advanced age and managed with oral antidiabetic therapy, with no history of early-onset or insulin-dependent diabetes. At age 10, in 1985, the patient presented with diabetes onset characterized by polyuria and polydipsia, blood glucose levels between 120 and 230 mg/dL, negative ketonemia, and glucosuria. At onset, her glycated hemoglobin (HbA1c) was 6.0% (42 mmol/mol), and pancreatic islet autoantibodies (ICAs) were negative in two separate determinations. HLA typing revealed DR3. Although C-peptide values are not available, the medical record indicates reduced pancreatic reserve. It is important to note that the term “reduced pancreatic reserve” should be interpreted with caution, as we do not have access to the specific analytical values for C-peptide and glucose from that time. She was included at the time in a randomized clinical trial under the diagnosis of type 1 diabetes mellitus, aimed at evaluating whether immunosuppressive treatment could induce diabetes remission [5]. She was allocated to the dual-treatment group and received azathioprine 75 mg daily and thymostimulin one intramuscular ampoule monthly for four years (from ages 10 to 14).

One year after the initiation of immunosuppressive therapy a glucagon test was performed to evaluate pancreatic reserve, with normal results (C-peptide 5 ng/ml with blood glucose of 154 mg/dL 30 min post-injection). Between ages 14 and 19, the patient was asymptomatic and had no other comorbidities. The patient did not require treatment with either oral antidiabetic drugs or insulin therapy from onset until the age of 19.

At age 19, cardinal symptoms of diabetes reappeared, with polyuria, polydipsia, an HbA1c of 9.1% (76 mmol/mol), and frank hyperglycemia above 200 mg/dL. Regarding the physical examination, the patient has a body mass index (BMI) of 20.2 kg/m². A repeat glucagon test confirmed insulinopenia (C-peptide < 0.8 ng/mL in all determinations). Screening for diabetic complications (retinopathy and nephropathy) was negative at this time. Additionally, elevated transaminases were observed at diabetes onset (AST 54 IU/L [5–40], ALT 77 IU/L [5–40], GGT 73 IU/L), with normal total bilirubin and alkaline phosphatase levels. An abdominal ultrasound revealed two simple right renal cysts without significant pathology.

Following onset, the patient has remained on insulin therapy to date. Glycemic control has varied, with excellent control (HbA1c 5.5-6.0% [37–42 mmol/mol]) during the first five years, followed by fluctuating levels (HbA1c 7.0-8.2% [53–66 mmol/mol]). Thirty years after diabetes onset, the patient has not developed diabetes-related complications. Autoimmunity screening was repeated in 2024 with negative results (anti-GAD65, anti-IA2, anti-ZnT8), and a C-peptide level of 0.24 ng/mL with blood glucose of 125 mg/dL.

In terms of comorbidities, liver function abnormalities observed during initial admission resolved during follow-up. However, in 2010, the patient again presented generalized pruritus accompanied by elevated AST, ALT, and GGT (64, 106, and 83 IU/L, respectively), alkaline phosphatase of 413 U/L (46–116), and normal total bilirubin (0.7 mg/dL). Liver autoimmunity tests were negative. Imaging studies, including abdominal ultrasound and magnetic resonance cholangiopancreatography, showed simple renal cysts without hepatic, gallbladder, or pancreatic abnormalities. Liver biopsy demonstrated minimal steatosis and nucleolar glycogenic degeneration. Pruritus was managed with cholestyramine and ursodiol, yielding partial improvement. Concurrently, persistent hypomagnesemia was documented (1.0-1.2 mg/dL, normal range 1.8–2.6 mg/dL), with normal levels of other electrolytes and a preserved renal profile. Gynecological evaluation for menorrhagia revealed a bicornuate uterus with multiple uterine fibroids. The patient did not exhibit renal function deterioration or hyperuricemia during follow-up, nor were neuropsychiatric abnormalities reported.

Ultimately, genetic testing identified a complete heterozygous deletion of the *HNF1B* gene. This pathogenic variant was incidentally discovered as part of a genetic panel designed to assess liver disorders, not initially targeted for diabetes symptoms. A 1.3 Mb deletion affecting the 17q12 chromosomal region, encompassing *ACACA* (*OMIM200350)*,* HNF1B (OMIM*189907), and *PIGW* (OMIM*610275), was confirmed through whole-exome sequencing (WES), which detected a copy number variation (CNV), and subsequently validated via array-comparative genomic hybridization (array-CGH). The genetic study did not reveal alterations in other OMIM-morbid genes. Although *PIGW* and *ACACA* are associated with autosomal recessive conditions, no clinical manifestations would be expected from their heterozygous deletion. The patient did not exhibit any symptoms or alterations associated with these genes.

Cascade testing could not be performed as the patient has no siblings or offspring, and genetic testing was not conducted on the parents at the time of diagnosis. 

## Conclusion

This case underscores the importance of considering genetic syndromes in patients presenting with atypical forms of diabetes, as the simplest and most common explanation is not always the correct one. When this patient was diagnosed, knowledge about monogenic diabetes was limited, leading to classifications that did not take these genetic forms into account, especially when the clinical presentation overlapped with features of autoimmune diabetes, as seen in this case. This could result in treatments that, while well-intentioned, were not fully aligned with the underlying condition, such as the immunosuppressive regimen administered in this instance.

The broad phenotypic spectrum associated with *HNF1B* variants, including renal, hepatic, and metabolic abnormalities, emphasizes the need for a high index of suspicion and a multidisciplinary approach to diagnosis and management. The phenotype of diabetes is also very broad, both in terms of age at onset and pancreatic reserve, as well as response to treatment. This variability is a critical point to emphasize, as it is a significant factor in understanding the variable expressivity of the disease. Therefore, syndromic genes, particularly *HNF1B*, should be included in gene panel testing for individuals with suspected monogenic diabetes, even if they present a pure diabetes phenotype rather than the classical syndromic presentation. 

In line with the current ISPAD clinical guidelines, all patients diagnosed with diabetes at pediatric age and with negative pancreatic autoantibodies should be tested for genes related to monogenic diabetes. It is worth noting that, in this case, the genetic diagnosis was delayed, despite the presence of clinical signs suggestive of an *HNF1B* defect—such as genitourinary abnormalities and altered liver enzymes—that were already evident years earlier. Identifying these signs earlier could have led to a faster genetic diagnosis, potentially avoiding treatments less suited to the underlying condition. 

This recommendation is crucial in order to prevent unnecessary treatments, as seen in this case, and to ensure that patients receive management best suited to their actual condition.

The variable expressivity observed in this case also justifies performing cascade testing, even if relatives do not appear to have the complete *HNF1B* syndrome. This approach allows for the identification of potentially affected individuals who may not yet show symptoms, enabling early intervention and more tailored management.

**Fig. 1:**
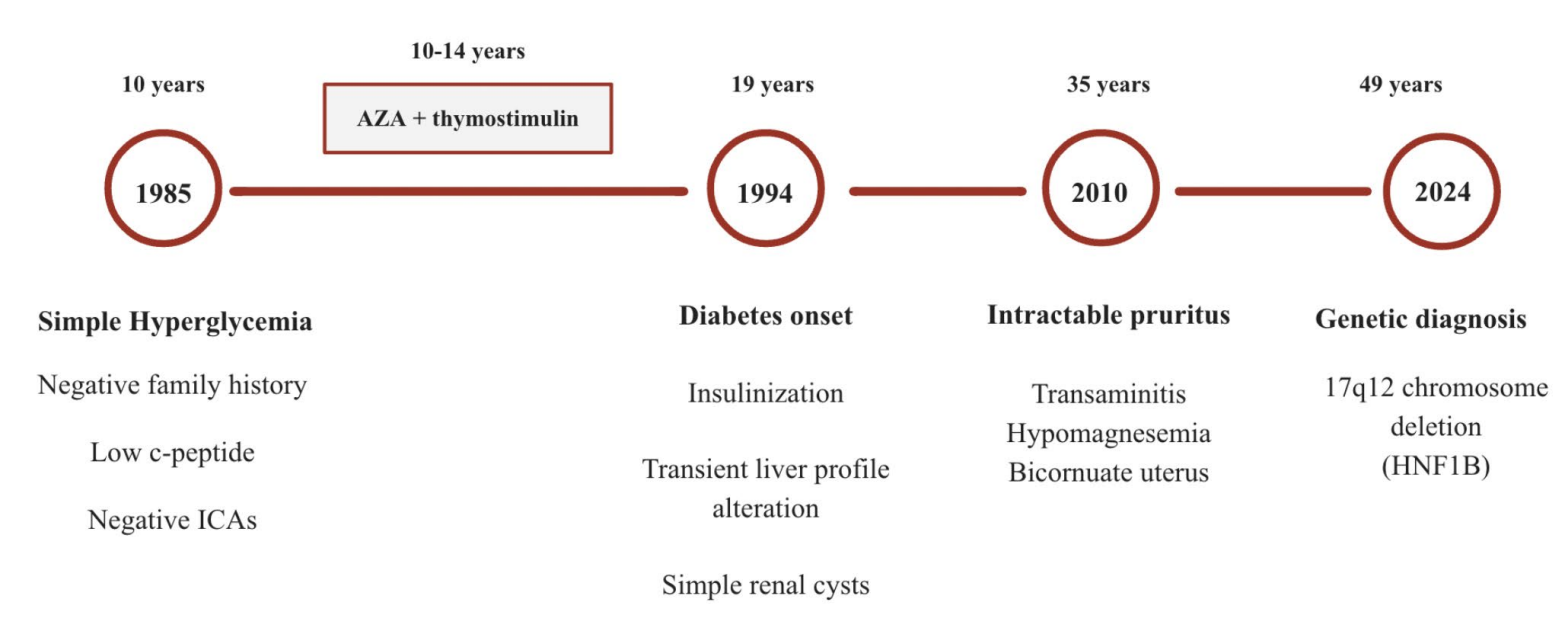
Clinical timeline illustrating the progression of events in a patient with *HNF1B*-associated diabetes. AZA: azathioprine. Key milestones include initial hyperglycemia with preserved C-peptide and negative autoimmunity (1985), onset of diabetes and renal cyst detection (1994), development of systemic manifestations such as transaminitis, hypomagnesemia, and bicornuate uterus (2010), and final genetic diagnosis of a 17q12 chromosome deletion involving HNF1B (2024)

Overall, this case contributes to the growing understanding of the clinical and genetic spectrum of *HNF1B*-associated conditions and underscores the value of genetic testing in atypical diabetes cases. Early diagnosis can lead to more personalized management strategies and the avoidance of unnecessary treatments (Fig. 1).
